# Safety of a fixed-dose combination of artesunate and amodiaquine for the treatment of uncomplicated *Plasmodium falciparum* malaria in real-life conditions of use in Côte d’Ivoire

**DOI:** 10.1186/s12936-016-1655-1

**Published:** 2017-01-03

**Authors:** Serge-Brice Assi, Yapo Thomas Aba, Jean Claude Yavo, Abouo Franklin Nguessan, N’cho Bertin Tchiekoi, Koffi Moïse San, Emmanuel Bissagnéné, Stephan Duparc, Valérie Lameyre, Mea Antoine Tanoh

**Affiliations:** 1 Institut Pierre Richet (IPR)/Institut National de Santé Publique (INSP), Bouaké, Ivory Coast; 2 Infectious and Tropical Diseases Department, Bouaké University Hospital, Bouaké, Ivory Coast; 3 Pharmacovigilance Unit, Medical Sciences, Felix Houphouët-Boigny, Abidjan, Ivory Coast; 4 Infectious and Tropical Diseases Unit, Treichville University Hospital, Abidjan, Ivory Coast; 5 National Malaria Control Programme, Abidjan, Ivory Coast; 6 Medicines for Malaria Venture, Geneva, Switzerland; 7 Sanofi Access to Medicines, Gentilly, France

**Keywords:** Malaria, Artesunate–amodiaquine, Côte d’Ivoire, Pharmacovigilance

## Abstract

**Background:**

In many malaria-endemic, sub-Saharan African countries, existing pharmacovigilance systems are not sufficiently operational to document reliably the safety profile of anti-malarial drugs. This study describes the implantation of a community-based pharmacovigilance system in Côte d’Ivoire and its use to document the safety of ASAQ Winthrop^®^ (artesunate–amodiaquine).

**Methods:**

This prospective, longitudinal, descriptive, non-comparative, non-interventional study on the use of artesunate–amodiaquine in real-life conditions of use was conducted in seven Community Health Centres of the Agboville district in Côte d’Ivoire. Twenty trained Health Centre employees and 70 trained community health workers were involved in data collection in the field. All patients with suspected uncomplicated falciparum malaria, seeking treatment at one of the participating Health Centres, and treated with artesunate–amodiaquine could be enrolled. Two visits were planned, one for inclusion at the Health Centre and a second at home, performed by a community health worker 3–10 days after the inclusion visit. Administration of artesunate–amodiaquine was unsupervised. Adverse events (AEs) were documented at the home visit or during any unexpected visit to the Health Centre or to the hospital and coded and adjudicated by a local pharmacovigilance committee. Symptoms suggestive of hepatic failure, severe neutropaenia, extrapyramidal disorders and retinopathy were considered a priori as AEs of special interest.

**Results:**

Some 15,228 malaria episodes in 12,198 patients were evaluated; 2545 AEs were documented during 1978 malaria episodes (13.0%). The most frequently observed events were asthenia (682 cases), vomiting (482 cases) and somnolence (174 cases). Most reported AEs were of mild or moderate intensity and resolved without corrective treatment. One-hundred and five (105) AEs reported during 100 episodes (0.7%) were considered as serious. Three serious cases of transient extrapyramidal disorders, identified as AEs of special interest were reported in three patients.

**Conclusion:**

The fixed dose artesunate–amodiaquine combination ASAQ Winthrop^®^ for the unsupervised treatment of uncomplicated falciparum malaria under real-life conditions of care in Côte d’Ivoire is well tolerated. The study emphasizes the interest of involving properly trained community health workers to collect pharmacovigilance data in the field in order to document rare AEs.

**Electronic supplementary material:**

The online version of this article (doi:10.1186/s12936-016-1655-1) contains supplementary material, which is available to authorized users.

## Background

Since 2001, artemisinin-based combination therapy (ACT) is recommended by the World Health Organization (WHO) as the mainstay of treatment of uncomplicated falciparum malaria [1, 2]. Between 2004 and 2007, a fixed-dose, combined formulation containing artesunate (AS) and amodiaquine (AQ) in a single tablet, was developed by Sanofi in partnership with Drugs for Neglected Disease initiative (DND*i*) (ASAQ Winthrop^®^) [3]. A large number of clinical trials [4] have shown that the ASAQ fixed-dose combination is effective in clearing *Plasmodium falciparum* parasites from infected individuals [5–13].

In Côte d’Ivoire, 43% of consultations in Health Centres are motivated by suspected malaria and effective management is a major public health issue [14]. A National Malaria Control Programme (NMCP) was established in 1996 with a number of specific policy areas covering prevention, treatment, education, and infrastructure. Notably, in 2007, systematic provision of ACT in local Health Centres (HCs) was adopted, and free or fixed combinations of AS and AQ were proposed as first-line treatment, with artemether–lumefantrine combinations as second-line treatment for uncomplicated falciparum malaria. At the time of the introduction of ASAQ Winthrop^®^, Sanofi and the DND*i* wanted to ensure that appropriate post-marketing data were available as quickly as possible regarding the safety and effectiveness of this anti-malarial treatment in the field. Existing pharmacovigilance systems in many endemic sub-Saharan African countries were not sufficiently operational to document reliably the safety profile of this drug. For this reason, a deployment monitoring plan for ASAQ Winthrop^®^ was designed to provide quality efficacy and safety data through a variety of pro-active studies, each providing different types of data. These studies have ranged from randomized, comparative, clinical trials in a limited number of patients treated under well-controlled conditions, to longitudinal, randomized cohorts to assess safety and efficacy in repeated administration, and finally to large-scale studies assessing the drug’s safety in ‘real-life’ conditions [15]. As part of this programme, the Agboville health district in Côte d’Ivoire was chosen as the location of a large, naturalistic, observational study to collect safety data on ASAQ Winthrop^®^ treatment under conditions of real-life use.

Before implementation of this study, a pharmacovigilance training programme for selected HC employees and community health workers (CHWs) was set up in order to optimize data collection. This programme was implemented through a partnership between Côte d’Ivoire Health Ministry, National Research Institutes (Institut Pierre Richet/Institut National de la Santé Publique, Côte d’Ivoire), University Departments of Côte d’Ivoire, DND*i*, Medicines for Malaria Venture (MMV) and Sanofi.

The primary objective of the study was to determine the safety of ASAQ Winthrop^®^ in real-life conditions of care in Côte d’Ivoire through the collection of clinical safety data. It was designed to evaluate at least 15,000 treated malaria episodes in order to permit detection of rare adverse events (AEs), which may have escaped detection in previous smaller, interventional, clinical trials. Secondary objectives included evaluation of the safety profile of ASAQ Winthrop^®^ in sub-groups of patients defined according to parasitaemia status, and to assess patient compliance.

## Methods

This was a prospective, longitudinal, descriptive, non-comparative, non-interventional study on the use of ASAQ Winthrop^®^ in real-life conditions of use, conducted in HCs of the Agboville district, located approximately 80 km north of Abidjan, Côte d’Ivoire, between February 2010 and October 2013. It was originally planned to perform the study over a 2-year period in only four HCs, but this was extended to 3 years and seven centres due to slow enrolment and to the outbreak of the 2010–2011 second Ivoirian Civil War, which threatened to disrupt the conduct of the study.

Over the course of the study, 20 trained employees working in HCs who receive patients for consultation and 70 trained field CHWs who visit patients in their homes were involved in data collection, and regularly supervised by the investigator team. To allow proper collection of safety data, two training sessions were organized for all participating HC employees and CHWs, once prior to implementation, and once during the study. This training related to the fundamental principles of pharmacovigilance, specific study procedures for collecting safety data and recognition of symptoms evoking AEs of special interest (AESI) for ASAQ Winthrop^®^. The CHWs were provided with bicycles and the HCs with motorcycles to enable all patients to be visited in their territory rapidly and effectively.

### Implementing partners

This study was implemented through a partnership that included the NMCP, the pharmacovigilance unit of the Pharmacology Department of Medical Sciences of the University of Abidjan, and the pharmacovigilance director of the Drug and Pharmacy Authority of the Côte d’Ivoire. Local community leaders were also involved to ensure successful implementation of the study in their territory. The study was coordinated by SBA, with the assistance of two co-investigators, an Independent Scientific Committee, a Pharmacovigilance Committee, a local Scientific Committee and a local Pharmacovigilance Committee. The role of the different study partners in the collection and analysis of the data is illustrated in Additional file 1.

### Eligibility criteria

All patients with suspected uncomplicated malaria who presented to the study centres, were able to take oral medication, were prescribed ASAQ Winthrop^®^ and who provided signed informed consent were eligible. During the course of the study in May 2011, national guidelines for malaria management changed from malaria diagnosis based on clinical suspicion only to a requirement for confirmation by a positive rapid diagnostic test (RDT) for antibodies against histidine-rich protein 2. The inclusion criteria for the study were modified accordingly. Exclusion criteria related to known allergy to AS or AQ, known pregnancy, severe malaria, visual disorders suggestive of retinopathy, and hepatic or haematological disorders occurring during previous treatment with AQ.

### Study design and procedures

All patients with suspected uncomplicated falciparum malaria seeking treatment at one of the participating HCs and treated with ASAQ Winthrop^®^ could be enrolled. All patients attending a participating HC were entered into the registry of the HC and all patients presenting suspected malaria were recorded in a specific screening registry, which documented date of visit, age and gender of the patient, village or district of origin, and, after May 2011, the result of RDT. If the patient met the selection criteria to participate in the study, and provided informed consent, a patient number was generated and a case report form (CRF) established; otherwise, the reason for non-participation was documented in the registry. Two visits were scheduled per patient, a first visit for inclusion at the HC and a second follow-up visit at home performed by a CHW 3–10 days after the initial visit. Additional unplanned visits for safety issues at the HC within 28 days of treatment initiation were also documented.

At the inclusion visit, demographic features (age, gender, weight), concomitant diseases, concomitant medication, previous use of traditional medication or anti-malarial drugs in the previous 2 weeks, and clinical features were documented. A finger-prick blood sample was taken using a vaccinostyle for a thick blood smear, which was to be read later centrally. Patients who met inclusion criteria were provided with a blister of ASAQ Winthrop^®^ covering the full 3-day treatment. At the follow-up visit, CHWs asked patients whether any clinical signs had appeared or worsened since the inclusion visit and, if this was the case, documented the signs or symptoms reported, date of onset, intensity, and outcome. In the case of detection of an AE requiring medical attention, the patient was addressed to the HC where further information was collected on seriousness, severity and management. CHWs counted and documented any ASAQ Winthrop^®^ tablets remaining in the blister pack provided at the HC. Information was collected on any other treatments taken since the inclusion visits, including treatments prescribed at the HC and traditional medicines. Patients were informed of the need to re-contact the HC in case of onset or worsening of any symptoms appearing after the visit of the CHW.

### Treatment

ASAQ was prescribed to eligible patients as oral tablets (ASAQ Winthrop^®^) to be taken once a day for 3 days using the recommended treatment regimen. Administration of ASAQ Winthrop^®^ was unsupervised. The dose was adjusted according to patient’s age, within four age/weight ranges as specified in the ASAQ Winthrop^®^ prescribing information.

### Determination of parasitaemia

Two thick blood smears were prepared from the blood sample taken at the inclusion visit and stained with a May-Grünwald-Giemsa solution for subsequent centralized reading to determine parasite density. One slide was sent to Abidjan for reading and the second was kept in the HC as back-up. All blood smears were provided to a central laboratory (Institut Pierre Richet, Abidjan and Bouaké, Côte d’Ivoire) where they were read by qualified personnel according to standard laboratory procedures. Parasite density was determined by reading 200 high-powered fields on the thick blood smear and counting the number of asexual parasites and white blood cells. Slides were considered negative if no parasite was detected after reading 200 high-powered fields. The presence and density of gametocytes was also determined. A randomly selected 10% sample of the thick smears was double-read for quality control.

### Safety evaluation

Safety was evaluated through documentation of AEs. All AEs documented at the follow-up visit or during a subsequent visit to the HC were coded using the Medical Dictionary for Regulatory Activities (MedDRA) version 17.0 and classified by severity and by seriousness. All AEs were reviewed centrally by the local pharmacovigilance committee for attribution of causality, according to a decisional algorithm based on French [16] and WHO [17] methods. These two methods differ in being more (French) or less (WHO) structured and in the criteria considered (such as rechallenge, previous exposure or concomitant medication) [18]. Hospital staff in Agboville were asked to report to the investigator team any cases of hepatotoxicity or agranulocytosis occurring in study participants who attended hospital. Any corrective treatment prescribed at the HC or at hospital was documented. Symptoms suggestive of hepatic failure (such as jaundice or severe pruritus), severe neutropaenia (such as mouth ulcerations), extrapyramidal disorders (abnormal movements, tongue protrusion) and retinopathy (blurred vision) were considered a priori as AESIs, and these were handled in the same way as serious AEs (SAEs). Procedures describing the management of identified SAEs or AESI by the HC personnel were validated by the infectious disease department of Treichville Hospital in Abidjan and outcome followed up with the relevant HC. All SAEs and AESIs were immediately communicated to the National Pharmacovigilance centre for Côte d’Ivoire and reviewed by the study Pharmacovigilance Committee. All relevant important information from this review was then forwarded to the members of the Independent Scientific Committee. Information on other AEs was communicated to the National Pharmacovigilance centre at regular intervals (approximately two-monthly) following causality review.

In the case of pregnancy being identified by midwives of the HCs in patients who had taken ASAQ Winthrop^®^, women were followed up until delivery and the outcome reported to the Sanofi pharmacovigilance department.

### Statistical analysis

This was not a hypothesis-testing study and no target sample size was determined a priori. The study was intended to enrol all patients attending a participating malaria treatment centre and prescribed ASAQ Winthrop^®^. It was estimated that documentation of at least 15,000 malaria episodes should allow identification of rare AEs occurring with an incidence of one in 5000. Safety analysis was performed on episodes experienced by all patients who had provided signed informed consent and taken at least one dose of ASAQ Winthrop^®^.

### Quality control

Medication blisters were collected at the follow-up visits by CHWs and then filed in the CRF. In order to assess compliance, the number of remaining tablets was recorded in the CRF. Completion of all CRFs and the data therein, including verification of the number of remaining tablets, were checked by a monitor at the time of collection. The investigator team visited and inspected all participating HCs on a quarterly basis to ensure compliance with the study protocol. As a quality control measure, approximately 10% of the CRFs collected in each HC were checked against the centre’s patient registry. When inconsistencies were detected, the proportion of CRFs to be checked was increased, and corrective actions were implemented.

## Results

### Patient disposition

**Fig. 1:**
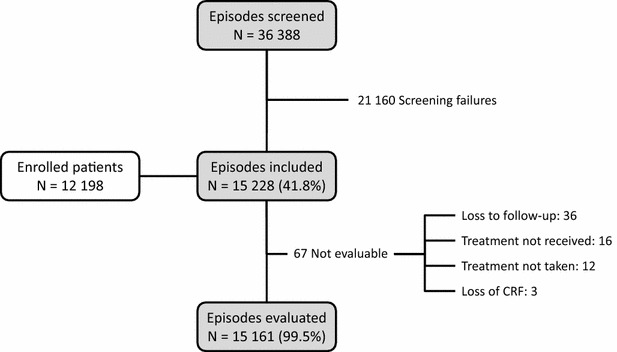
Patient disposition. *CRF* case report form

Patient disposition is illustrated in Fig. 1. Overall, 36,388 malaria episodes in patients attending a participating centre were screened for eligibility. Of these, 15,228 episodes (41.8%) in 12,198 patients met the eligibility criteria. The principal reasons for non-eligibility were a negative RDT (32% of attacks screened), severe malaria (19%) and patients who could not be followed up (17%). Of the 12,198 enrolled patients, 10,294 (84.4%) experienced one identified episode of malaria, 1258 (10.3%) two episodes, 374 (3.1%) three episodes and 272 (2.2%) patients more than three episodes over the study period. Sixty-seven of the episodes in 67 patients (0.5%) could not be assessed after treatment and were excluded from the safety population. The reasons for non-assessment were loss to follow-up (N = 36; 53.7%), treatment not received by the patient (N = 16; 23.9%), treatment not taken by the patient (N = 12; 17.9%) and loss of the CRF (N = 3; 4.5%).

Of the 15,228 eligible episodes, 51.5% occurred during the rainy season and 48.5% during the dry season. The mean number of episodes was 1.2 ± 0.7 per patient (median 1; range 1–11), during the rainy season and 0.6 ± 0.7 episodes per patient (median 1; range 1–6) during the dry season.

### Patient characteristics

Patient characteristics at inclusion are presented by malaria episode in Table 1. Nearly two-thirds of these episodes (64.7%) occurred in patients under 6 years of age (median age 3 years) and the gender ratio was close to unity. All episodes with available data (N = 15,225) were associated with signs or symptoms consistent with malaria, most frequently fever (87.3%); other symptoms were documented for <20% of episodes. Some of these symptoms were reported more often as signs of malaria rather than of concomitant disease, notably headache (19.7 vs 0.1%), vomiting (15.2 vs 1.4%), asthenia (9 vs 0.2%), and joint ankylosis/arthralgia (8.7 vs 0.2%), while others were more often reported as signs of concomitant disease (cough/productive cough: 21.6 vs 0.1%) and, more surprisingly, anaemia (20.7 vs 1.8%). No difference was observed in the nature or frequency of presenting symptoms between episodes occurring during the rainy season and those occurring during the dry season.

**Table 1:** Characteristics of malaria episodes

	N = 15,228
Age (years)	*N* = *15,225*
Mean ± SD	8.9 ± 13.3
Median [range]	3 [1–90]
Age by class	*N* = *15,225*
≤1 month	2 (<0.01%)
2–11 months	2111 (13.9%)
1–5 years	7740 (50.8%)
6–13 years	2581 (17.0%)
≥14 years	2791 (18.3%)
Gender	*N* = *15,225*
Female	7582 (49.8%)
Male	7643 (50.2%)
Weight (kg)	*N* = *15,221*
Mean ± SD	21.6 ± 18.9
Median [range]	13 [2–116]
Weight by class (kg)	*N* = *15,221*
<4.5	35 (0.2%)
4.5 to <9	2704 (17.8%)
9 to <18	7241 (47.6%)
18 to <36	2278 (15.0%)
≥36	2963 (19.5%)
Clinical signs and symptoms^a^	*N* = *15,225*
Fever	13,294 (87.3%)
Hyperthermia	1608 (10.6%)
Asthenia	1373 (9.0%)
Chills	244 (1.6%)
Headache	3005 (19.7%)
Dizziness	228 (1.5%)
Vomiting	2316 (15.2%)
Diarrhoea	589 (3.9%)
Abdominal pain	316 (2.1%)
Decreased appetite/anorexia	2901 (19.1%)
Joint ankylosis	1223 (8.0%)
Anaemia	273 (1.8%)
Parasitaemia	*N* = *14,634*
Positive thick blood smears	11,192 (76.5%)

The N numbers in italics refer to the number of episodes for which the variable of interest was documented

^a^Frequency counts correspond to number of episodes, since the same patient may have experienced more than one episode. Certain symptoms documented from self-report, such as pain, headache and dizziness, could not be ascertained in young children

A readable thick blood smear was obtained for 14,634 malaria episodes (96.1%) and these were positive for *P. falciparum* in 76.5% of cases. The diagnosis of malaria was more frequently confirmed from the blood smears (*p* < 0.001) for episodes occurring during the rainy season (75.3% of episodes) compared to the dry season (71.6%). No difference was observed in the nature or frequency of presenting symptoms between patients with or without parasites on blood smear. Administration of traditional medications prior to enrolment was documented in 16.5% of the episodes. At least one concomitant treatment was prescribed at inclusion in almost all episodes (99.5%), most commonly antipyretics (90.5%), systemic antibiotics (37.2%), antiparasitic medicines (28.6%), and iron preparations (25.7%).

### Treatment compliance

As assessed by questioning and by counting remaining tablets, ASAQ Winthrop^®^ was taken completely for 14,736 out of 15,161 malaria episodes [97.2% (95% CI 96.9–97.5%)]. The most frequent reason for non-compliance was treatment discontinuation due to the occurrence of an AE (180/425 cases). The dose of ASAQ Winthrop^®^ prescribed was appropriate for the patient’s age or weight in the majority of episodes (98.6% for age and 86.1% for weight).

### Adverse events

All but 36 episodes benefitted from a follow-up home visit by a CHW. Overall, 2545 AEs were documented during 1978 of 15,161 malaria episodes (13.0%) during the course of the study (Table 2). The most frequently observed AEs were asthenia (682 cases; 4.5%), vomiting (482 cases; 3.1%) and somnolence (174 cases; 1.1%). Malaria was reported as an AE during 39 episodes. Of these AEs, 105 (4.1%) reported during 100 episodes (0.7%) were considered serious. The most commonly observed SAEs were malaria cases (29 episodes; 0.2%), anaemia (28 episodes; 0.2%) and gastroenteritis (7 episodes; <0.1%); no other SAE was reported more than five times; no SAE was considered as a new signal. The majority of AEs were mild (1449 events during 1225 episodes; 56.9%) or moderate (942 events during 771 episodes; 37%) in intensity. Of the severe AEs (6.1%), the most frequently reported were malaria (N = 26; 0.2%), anaemia (N = 29; 0.2%), asthenia (N = 17; 0.1%), vomiting (N = 13; 0.1%) and pyrexia (N = 9; 0.1%).

**Table 2:** Overview of adverse events

	Number of adverse events	Number of episodes	Incidence
Any adverse event	2545	1978	13.0% [12.5%; 13.6%]
Serious adverse events	105	100	0.7% [0.5%; 0.8%]
Severe adverse events	154	126	0.8% [0.7%; 1.0%]
Adverse events of special interest	3	3	<0.1%
Fatal serious adverse events	24	22	0.2% [0.1%; 0.2%]
Potentially ASAQ-related adverse events	929	837	5.5% [5.2%; 5.9%]
Adverse events requiring corrective treatment	157	105	0.7% [0.6%; 0.8%]

Twenty-two deaths, none considered potentially related to the study drug, were reported. Three AESIs, all involving cases of extrapyramidal disorders, were reported in three patients. Two of these patients had also received *djanougbokpè* from a folk healer. This is a mixture of roots, leaves or bark of different plants with purgative properties, which is traditionally used for the treatment of malaria in West African societies. The exact composition of *djanougbokpè* may vary from one folk healer to another. The other patient received a Chinese herbal medicine whose composition was not identified on the packet. All cases resolved successfully following corrective treatment with intravenous diazepam. One of these patients was unintentionally rechallenged with ASAQ Winthrop^®^ during a subsequent malaria episode two months later without re-appearance of the symptoms.

Most AEs occurred early, during the 3 days of ASAQ Winthrop^®^ treatment. The mean time to onset of the AEs after the first administration of ASAQ Winthrop^®^ was 1.3 ± 1.9 days. Some of these events were recorded as AEs retrospectively at the time of the home visit, even though they had appeared soon after administration of ASAQ Winthrop^®^. The distribution of AEs according to the time of the home visit is presented in Additional file 2. More AEs were reported when the home visit by the CHW was made soon after treatment initiation, although some AEs were recorded even when the home visit was performed 10 days later. It should be noted that over 80% of the home visits were made between 3 and 6 days (inclusive) after treatment initiation.

Potentially ASAQ-related AEs (N = 929; 36.5% of all AEs) were reported during 837 episodes (5.5%). The most frequent of these are listed in Table 3. Only asthenia, somnolence, vomiting, and dizziness were reported at an incidence of over one case per 1000 episodes. Of these potentially ASAQ-related AEs, 11 (1.2%) were serious, of which seven were also rated as severe. These ASAQ-related SAEs are listed in Table 4. All potentially related SAEs resolved spontaneously or after treatment (the three cases of extrapyramidal disorder and one case of asthenia). In addition, 15 potentially related, non-serious AEs were rated as severe, principally asthenia (five cases), all of which resolved. Furthermore, eight non-serious, non-severe AEs had not resolved by the end of the observation period and a further five required corrective treatment. A full list of potentially ASAQ-related AEs that were serious, severe, unresolved, or that required treatment is provided in Additional file 3.

**Table 3:** Potentially ASAQ-related adverse events

	Number of adverse events	Number of episodes	Incidence^a^
Any potentially ASAQ-related adverse event	929	837	5.5%
Asthenia	410	410	2.7
Somnolence	134	134	0.9
Vomiting	89	89	0.6
Dizziness	84	84	0.6
Diarrhoea	21	21	0.1
Insomnia	13	13	0.1
Abdominal pain	12	12	0.1
Pruritus	26	26	0.2
Decreased appetite	28	28	0.2
Cough	13	13	0.1

Only individual adverse events reported during more than ten malaria episodes are listed in this table

^a^Calculated with respect to the 15,228 documented malaria episodes

**Table 4:** Treatment-related or fatal serious adverse events

SAEs considered related to ASAQ	11
Asthenia	4
Vomiting	3
Extrapyramidal disorders	3
Dyspnoea	1
Fatal SAEs (all considered unrelated)	24^a^
Malaria	8
Malaria with cough	1
Anaemia	4
Anaemia with sepsis	1
Pneumonia	1
Diarrhoea with dehydration	1
Peritonitis	1
Ascites	1
Hypoglycaemia	1
Encephalitis	2
Febrile convulsions	1

*SAE* serious adverse events

^a^22 patients died, two of whom presented two SAEs at the time of death (anaemia with sepsis in one patient and diarrhoea with dehydration in the other). A further patient presented with malaria (serious) and cough (non-serious) at the time of death

Regarding outcome, 2452 of the 2545 AEs resolved satisfactorily (96.3%) without sequelae. In addition, 39 AE were improving at the time of last follow-up. One patient presented a non-serious skin rash which left marks and scars. Twenty-four SAEs during 22 episodes (0.1%) were fatal and these are listed in Table 4. None of these fatal AEs was considered possibly related to treatment with ASAQ Winthrop^®^.

### Adverse events as a function of parasitaemia

The incidence of AEs was compared between malaria episodes with parasitaemia detected on thick blood smears, unconfirmed malaria episodes without detectable parasitaemia, and episodes without available parasitaemia results (poor quality of blood smear preparation, blood smear not performed or destroyed). No statistically significant differences in the incidence or nature of AEs, SAEs or potentially ASAQ-related AEs were observed between the three sub-groups (Table 5).

**Table 5:** Incidence of adverse events as a function of parasitaemia

	Positive parasitaemia	Negative parasitaemia	Undetermined parasitaemia	*p*
	N = 11,141	N = 3435	N = 585	
Any AE	12.9% [12.3%; 13.5%]	13.7% [12.5%; 14.8%]	11.8% [9.2%; 14.4%]	0.333
Any SAE	0.7% [0.5%; 0.8%]	0.6% [0.4%; 0.9%]	0.5% [0.0%; 1.1%]	0.905
Any potentially ASAQ-related AE	5.6% [5.2%; 6.1%]	5.4% [4.6%; 6.1%]	4.4% [2.8%; 6.1%]	0.423

The occurrence of adverse events is presented as the incidence rate (%) with its 95% confidence interval

## Discussion

This large, naturalistic, observational study was designed to monitor the safety of ASAQ Winthrop^®^ under conditions of every day care in malaria treatment centres in Côte d’Ivoire. The study was successfully implemented and the target sample size achieved. This illustrates the feasibility of such studies for monitoring anti-malarial treatments in West Africa and to complement ‘passive’ pharmacovigilance systems. The study population was principally composed of young children, with 65% of the sample being under 6 years of age, which reflects the epidemiology of malaria in many sub-Saharan African countries [19]. The relatively low rates of multiple infections over the course of the study can be attributed to the design of the study. Since this was not a longitudinal study aimed at investigating multiple malaria episodes, patients were not required to attend the same study HCs for consecutive malaria episodes. Compliance with treatment was high in spite of the absence of supervised administration of ASAQ Winthrop^®^. AEs were documented in 13% of >15,000 individual treated malaria episodes in >12,000 patients over a period of 3½ years. Overall, treatment with ASAQ Winthrop^®^ was found to be well tolerated and the nature of AEs reported was consistent with the known safety profile of ASAQ Winthrop^®^. Most reported AEs were not severe and resolved without the need for medical intervention.

In this study, a robust and structured reporting system was set up for AE reporting, with the HC staff systematically reporting safety observations in patients seen at HCs and a trained network of involved and motivated CHWs who were encouraged to report all AEs. Despite the high rate of follow-up home visits, the reporting rate of AEs in this study was lower than that reported in randomized clinical trials of ASAQ Winthrop^®^ in sub-Saharan Africa [7, 10–13]. This finding was not unexpected and suggests that community-based AE surveillance may be less sensitive than surveillance in the context of a clinical trial in an HC. In clinical trials, patients are expected to return to the HC every day for the early part of the study and then episodically up to 28 days following inclusion. At these visits, patients are actively questioned about the occurrence of AEs. This is likely to elicit more reports than when patients are asked only once by a CHW, potentially several days after any AE had occurred. However, in this study, dedicated home visits allowed a higher rate of collection of AEs than non-systematic, face-to-face contacts, and in particular with respect to unsolicited self-reporting by patients (ten reported AEs per 100,000 treatments according to a retrospective study of ASAQ combinations in Senegal [20]). Nonetheless, the nature of potentially ASAQ-related AEs was similar between the present study and previous randomized clinical trials [7, 10–13], suggesting that these are adequately detected in the community setting.

It is of note that the rate of AE reporting was highest when the interval between treatment initiation and the home visit was short and reduction of the mean interval before the home visit may increase the sensitivity of AE reporting. Although home visits were supposed to take place between 3 and 10 days following initiation of treatment, a small number of visits were made before 3 days, and documentation of SAEs was proportionally more frequent during these visits. It can be hypothesized that these early visits may have been triggered by the occurrence of AEs, leading to villagers contacting the CHW. Nevertheless, if follow-up is too early, there may be a risk of missing delayed effects, even if patients were strongly encouraged to re-contact their CHW in case of any problem. In these cases, a further later follow-up visit may be useful to ensure comprehensive documentation of all AEs.

Comparison of this study with previous cohort-event monitoring studies (CEM) of anti-malarial drugs is complicated by differences in study design. No previous observational CEM study has evaluated the incidence of AEs in patients treated specifically with an identified ASAQ fixed-dose combination tablet, and several previous studies have evaluated different anti-malarial drugs in the same study. The present study is also much larger than previous studies, which included <5000 patients (with the exception of one recent multinational study that also recruited >10,000 patients [21]). In addition, different methods of data collection have been used in these various CEM studies. For example, in an early (2006) CEM study of AEs in patients receiving anti-malarials in Ghana [22, 23], patients were followed up by telephone contact or voluntary return clinic visits; AEs were reported in 29% of 2381 patients. However, the study population was different from that of this study, being exclusively urban and predominantly adult (66.1% aged ≥13 years) and the anti-malarial treatment was not restricted to a specific anti-malarial medicine (43% of treatments were free or fixed combinations of AS and AQ, all brands considered). In a CEM study of patients treated with AS and AQ combinations (various free or fixed formulations over time) in rural Senegal, performed between 2001 and 2009 [24], patients were required to return to the HC every day for the 4 days following start of treatment and again after 4 weeks, where data on AEs were collected using a structured questionnaire. The rate of AEs reporting in the study (12%) is close to that observed in the present study in Côte d’Ivoire. Other CEM studies of ACT in Nigeria [25] and of artemether–lumefantrine (Coartem^®^) in Tanzania [26] also required patients to return to the HC or to contact a CHW. The study that is perhaps the closest to the present one in design is the INDEPTH study of a fixed-dose combination of dihydroartemisinin and piperaquine phosphate (Eurartesim^®^), conducted in four African countries between 2013 and 2014 [21], which included 11,097 patients with RDT-confirmed *P. falciparum* infections who were followed up by home visits from a CHW or by telephone. More recently, a study in Ghana of patients prescribed ACT [27] has compared telephone contact and home visits by a CHW and found that the former method elicited more AEs and was less expensive to implement. On the other hand, a Nigerian study with telephone monitoring which enrolled patients buying anti-malarials in a pharmacy reported that, for many patients, telephone contact was not maintained over time [28].

Overall, 929 AEs potentially related to ASAQ Winthrop^®^ were reported during 837 episodes (5.5%). The most frequent of these potentially related AEs were asthenia, somnolence, vomiting, and dizziness, with an incidence of >0.5%. All these are known adverse drug reactions to ASAQ Winthrop^®^ and listed as such in the prescribing information. No cases of retinopathy, symptomatic leukopaenia or symptoms suggestive of serious abnormalities in liver function, which are characterized AEs associated with amodiaquine, were observed. However, no laboratory blood testing was performed, so asymptomatic leukopaenia or perturbations of liver function cannot be ruled out. The incidence of SAEs (0.7%) and severe AEs (0.8%) was low and, of the 22 deaths reported in the study, none was considered potentially related to ASAQ Winthrop^®^. The nature and frequency of SAEs were consistent with previous clinical trials [7, 10–13]. These findings suggest that SAEs are not under-reported in the naturalistic setting of this study and that the pharmacovigilance procedures implemented by the NMCP should be adequate to monitor the safety of extensive use of ASAQ Winthrop^®^ in Côte d’Ivoire.

The large sample size of the study (>15,000 malaria episodes) was chosen in order to allow identification of rare AEs occurring with an incidence of one in 5000. In this context, three cases of moderate extrapyramidal disorders (incidence of 0.02%) were recorded and considered as possibly drug-related. All cases resolved without sequelae after treatment with diazepam. Causality could not be proven since all patients had also taken non-prescribed traditional medications; furthermore, one patient was re-challenged with ASAQ Winthrop^®^ without recurrence of symptoms. Prior to the initiation of the study, the study sponsor became aware of poorly characterized pharmacovigilance reports of extrapyramidal symptoms in patients taking amodiaquine or artesunate and amodiaquine combinations of different origins and of unknown causality. This was the reason why extrapyramidal symptoms were listed in the study protocol as an AESI, in an effort to better characterize any potential association. The identification of three characterized cases in this study prompted the Marketing Authorization Holder for ASAQ Winthrop^®^ to carry out a comprehensive assessment of the association of extrapyramidal disorders with ASAQ Winthrop^®^ or other anti-malarial drugs in 2012, which led to extrapyramidal disorders being listed as adverse drug reactions in the prescribing information for ASAQ Winthrop^®^. Around the same time, an analysis was published of some of the earlier pharmacovigilance reports documented in the WHO global pharmacovigilance database (VigiBase™) [29], and there have been further subsequent reports of extrapyramidal symptoms in patients taking artemisinin and amodiaquine combinations elsewhere in Africa [23, 24, 30].

At the beginning of the study, national malaria treatment recommendations did not require confirmation of malaria diagnosis by a blood test, and treatment could be initiated on the basis of clinical suspicion only. However, since malaria symptoms are relatively non-specific, this policy exposed patients to unnecessary anti-malarial treatment with a potential risk of toxicity, and was changed during the course of the study in 2011, such that laboratory confirmation of the diagnosis was required. Nonetheless, given the high sensitivity of RDT, false positives may still occur in patients with fluctuating low parasite densities or carrying residual parasite proteins from a previous infection [31–33], in particular in high transmission areas such as Côte d’Ivoire. However, even before the requirement for RDT, a high rate of biological confirmation of suspected malaria was observed (82.6%). This is at the high end of the range of published studies of the accuracy of clinical diagnosis (40–80%) [34], indicating the quality of clinical assessment by trained and supervised health workers. Indeed, all the HC workers received training in the differential diagnosis of malaria before the study started, and diagnostic practice in each HC was supervised by an experienced tropical medicine specialist who visited the HC every one to three months. The AE profile of ASAQ Winthrop^®^ observed in the study did not differ between treated patients with RDT-confirmed malaria and in patients without identified parasitaemia.

Although treatment intake was unsupervised, the rate of reported compliance based on tablet count by CHWs at home visits was high (97.2%). It is possible that the fact that patients were aware that CHWs would be making home visits encouraged compliance (or hiding of tablets). For some patients, a home visit was made during the treatment period, when the CHW could check that the treatment was being taken correctly. Nonetheless, any such bias is likely to be limited and the good observed compliance rate may reflect the ease of administration of ASAQ Winthrop^®^, with a single intake of a low number of tablets (one or two) per day. In addition, these tablets can be either swallowed or dissolved in a small amount of water for people unable to swallow. The good compliance of ASAQ Winthrop^®^ use with prescription recommendations at HCs should be noted, with the prescribed dose being correctly adjusted for a patient’s age in 98.6% of episodes.

This study has a number of strengths and limitations. The principal strengths relate to the large number of patients included and the naturalistic treatment setting. The findings should thus be generalizable to every day malaria treatment practice in Côte d’Ivoire. In particular, the study included essentially all patients with suspected malaria treated with ASAQ Winthrop^®^ in the Agboville region during the study period, which covered all seasons of the year.

The principal limitation related to the lack of systematic ascertainment of reported AEs by a physician. Safety evaluation was limited to clinical observation, and laboratory testing was only performed on an ad hoc basis. In consequence, cases of leukopaenia or impaired liver function may have gone undetected if these were asymptomatic. In addition, treatment with ASAQ Winthrop^®^ was unsupervised, which means that certain patients may not have respected the recommended dosage regimen. Finally, a large proportion of patients were prescribed or had taken other medications at the same time as ASAQ Winthrop^®^ treatment, rendering unambiguous determination of causality very difficult. However, these limitations are necessary consequences of the naturalistic study design.

This type of study may help to extend awareness of the use of ACT for malaria treatment and on the importance of pharmacovigilance reporting by staff of local HCs and by CHWs, and thus contribute to effective long-term safety monitoring in the NMCP in Côte d’Ivoire. A high level of communication was maintained between the different partners in the network, which ensured that CHWs and local HC staff received information and training from the study coordinators whenever the need arose. Following this study, the NMCP of Côte d’Ivoire signed an agreement with the Drug and Pharmacy Board, which states that the latter should undertake training of healthcare workers in pharmacovigilance and analysis of pharmacovigilance report forms.

This study underlines the added value of systematic involvement of CHWs in collecting pharmacovigilance data on anti-malarial drugs in a community setting in sub-Saharan Africa, at least when new therapeutic programmes are being implemented. Although the WHO has emphasized the need for post-approval safety information on new medicines in African countries [35], this is particularly challenging in settings where spontaneous reporting of adverse drug reactions is low and there is no tradition of systematic pharmacovigilance reporting [36]. Studies such as this may be an effective and pragmatic way to respond to this challenge and to build local capacity in pharmacovigilance.

## Conclusion

This large, naturalistic study of the tolerability of ASAQ Winthrop^®^ for the unsupervised treatment of uncomplicated falciparum malaria under everyday conditions of care in Côte d’Ivoire demonstrated that the safety of the drug was consistent with what had been seen in the clinical trials, allowed the identification of extrapyramidal disorders as rare AEs leading to a change in prescribing information, and emphasized the value of involving properly trained CHWs to collect pharmacovigilance data in the field.

## Additional files

**Additional file 1.** Data reporting flow and responsibilities.

**Additional file 2.** Adverse-event reporting as a function of the interval between treatment initiation and the home visit.

**Additional file 3.** Overview of adverse events potentially related to ASAQ rated as serious, severe, unresolved, or requiring treatment.

## Abbreviations

ACT: artemisinin-based combination therapy
AE: adverse event
AESI: adverse event of special interest
AQ: amodiaquine
AS: artesunate
ASAQ: artesunate–amodiaquine fixed combination
CEM: cohort-event monitoring
CHW: community health worker
CRF: case report form
DND*i*: drugs for neglected disease *initiative*
HC: Health Centre
MedDRA: medical dictionary for regulatory activities
MMV: medicines for malaria venture
NMCP: National Malaria Control Programme
RDT: rapid diagnostic test
SAE: serious adverse event
WHO: World Health Organization
