# Supplementary figures and images for: Safety of a fixed-dose combination of artesunate and amodiaquine for the treatment of uncomplicated Plasmodium falciparum malaria in real-life conditions of use in Côte d’Ivoire
Source: Malar J. 2017 Jan 3;16:8. doi: 10.1186/s12936-016-1655-1 (PMC5209945; doi:10.1186/s12936-016-1655-1)

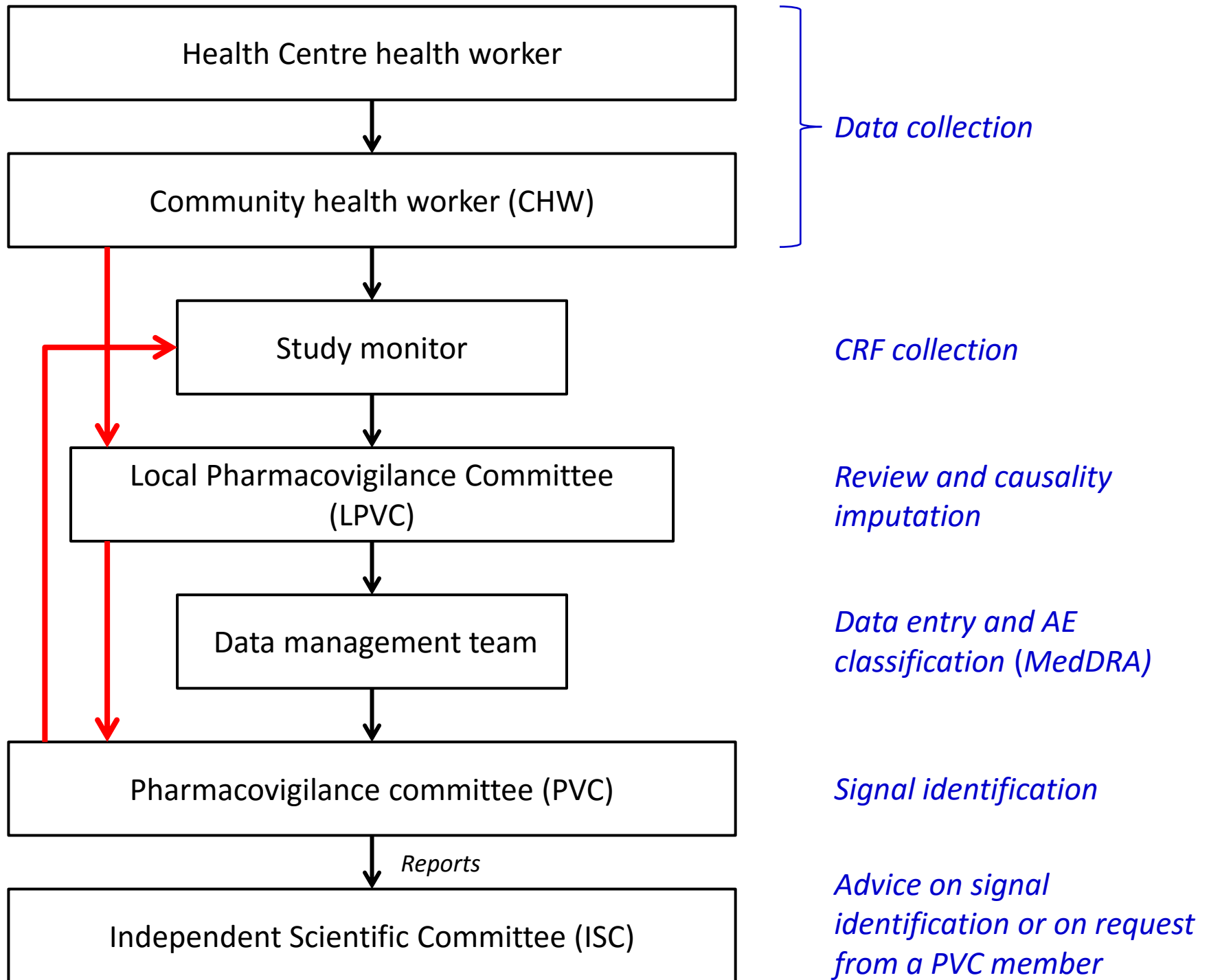

Supplement: Supplementary file 1 — Additional file 1. Data reporting flow and responsibilities. [file 12936_2016_1655_MOESM1_ESM.pdf]
