# Supplementary material for: Safety of a fixed-dose combination of artesunate and amodiaquine for the treatment of uncomplicated Plasmodium falciparum malaria in real-life conditions of use in Côte d’Ivoire
Source: Malar J. 2017 Jan 3;16:8. doi: 10.1186/s12936-016-1655-1 (PMC5209945; doi:10.1186/s12936-016-1655-1)
Supplement: Supplementary file 2 — Additional file 2. Adverse-event reporting as a function of the interval between treatment initiation and the home visit. [file 12936_2016_1655_MOESM2_ESM.docx]

Additional file 2 Adverse-event reporting as a function of the interval between treatment initiation and the home visit

|  | Date of follow-up visit | | | |
| --- | --- | --- | --- | --- |
|  | 0-2 days  (N=96) | 3-6 days  (N=12,312) | 7-10 days  (N=2,444) | >10 days  (N=309) |
| *Any adverse event* |  |  |  |  |
| N | 43 | 1 585 | 328 | 22 |
| % during period^1^ | (44.8%) | (12.9%) | (13.4%) | (7.1%) |
| % in class of AE^2^ | (2.2%) | (80.1%) | (16.6%) | (1.1%) |
| *Serious adverse events* |  |  |  |  |
| N | 20 | 62 | 14 | 4 |
| % during period^1^ | (20.8%) | (0.5%) | (0.6%) | (1.3%) |
| % in class of AE^2^ | (20.0%) | (62.0%) | (14.0%) | (4.0%) |
| *Adverse events of special interest* |  |  |  |  |
| N | 2 | 1 | None | None |
| % during period^1^ | (2.1%) | (0.0%) | - | - |
| % in class of AE^2^ | (66.7%) | (33.3%) | - | - |
| *Potentially ASAQ-related adverse events* |  |  |  |  |
| N | 14 | 672 | 144 | 7 |
| % during period^1^ | (14.6%) | (5.5%) | (5.9%) | (2.3%) |
| % in class of AE^2^ | (1.7%) | (80.3%) | (17.2%) | (0.8%) |
| *Adverse events requiring medical intervention* |  |  |  |  |
| N | 5 | 72 | 25 | 3 |
| % during period^1^ | (5.2%) | (0.6%) | (1.0%) | (1.0%) |
| % in class of AE^2^ | (4.8%) | (68.6%) | (23.8%) | (2.9%) |

^1^ Proportion of episodes for which an adverse event was reported (% by column)

^2^ Proportion of all adverse events occurring during the specified time period (% by row)
