# Supplementary material for: Safety of a fixed-dose combination of artesunate and amodiaquine for the treatment of uncomplicated Plasmodium falciparum malaria in real-life conditions of use in Côte d’Ivoire
Source: Malar J. 2017 Jan 3;16:8. doi: 10.1186/s12936-016-1655-1 (PMC5209945; doi:10.1186/s12936-016-1655-1)
Supplement: Supplementary file 3 — Additional file 3. Overview of adverse events potentially related to ASAQ rated as serious, severe, unresolved, or requiring treatment. [file 12936_2016_1655_MOESM3_ESM.docx]

Additional file 3

**Overview of adverse events potentially related to ASAQ rated as serious, severe, unresolved, or requiring treatment**

| Preferred term | Serious | Severe | Resolved | Treated |
| --- | --- | --- | --- | --- |
| Asthenia | Yes | Yes |  | Yes |
| Asthenia | Yes | Yes |  |  |
| Asthenia | Yes | Yes |  |  |
| Asthenia | Yes | Yes |  |  |
| Dyspnoea | Yes | Yes |  |  |
| Vomiting | Yes | Yes |  |  |
| Vomiting | Yes | Yes |  |  |
| Vomiting | Yes |  |  |  |
| Extrapyramidal disorder | Yes |  |  | Yes |
| Extrapyramidal disorder | Yes |  |  | Yes |
| Extrapyramidal disorder | Yes |  |  | Yes |
| Asthenia |  | Yes |  | Yes |
| Asthenia |  | Yes |  |  |
| Asthenia (worsened) |  | Yes |  |  |
| Asthenia (worsened) |  | Yes |  |  |
| Asthenia (worsened) |  | Yes |  |  |
| Joint ankyloses (worsened) |  | Yes |  |  |
| Joint ankyloses (worsened) |  | Yes |  |  |
| Abdominal pain |  | Yes |  |  |
| Dizziness |  | Yes |  |  |
| Dizziness |  | Yes |  |  |
| Palpitations |  | Yes |  |  |
| Pruritus |  | Yes |  |  |
| Insomnia |  | Yes |  |  |
| Hypersomnia |  | Yes |  |  |
| Vomiting |  | Yes |  | Yes |
| Vesicular rash |  |  | No |  |
| Generalized rash |  |  | No |  |
| Vomiting |  |  | No |  |
| Dysstasia |  |  | No |  |
| Pruritus |  |  | No |  |
| Cough |  |  | No |  |
| Cough |  |  | No |  |
| Cough |  |  | No |  |
| Vomiting |  |  |  | Yes |
| Vomiting |  |  |  | Yes |
| Pruritus |  |  |  | Yes |
| Pruritus |  |  |  | Yes |
| Rash |  |  |  | Yes |
